# Safeguarding Ecosystem Services: A Methodological Framework to Buffer the Joint Effect of Habitat Configuration and Climate Change

**DOI:** 10.1371/journal.pone.0129225

**Published:** 2015-06-19

**Authors:** Tereza C. Giannini, Leandro R. Tambosi, André L. Acosta, Rodolfo Jaffé, Antonio M. Saraiva, Vera L. Imperatriz-Fonseca, Jean Paul Metzger

**Affiliations:** 1 Department of Ecology, Institute of Bioscience, University of Sao Paulo (USP), R. do Matao 321, 05508–090, Sao Paulo, Sao Paulo, Brazil; 2 Computation and Digital Systems, Engineering School, University of Sao Paulo (USP), Av. Prof. Luciano Gualberto 380, 05508–010, Sao Paulo, Sao Paulo, Brazil; 3 Vale Institute of Technology Sustainable Development, Rua Boaventura da Silva 955, 66055–090, Belém, Pará, Brazil; Monash University, AUSTRALIA

## Abstract

Ecosystem services provided by mobile agents are increasingly threatened by the loss and modification of natural habitats and by climate change, risking the maintenance of biodiversity, ecosystem functions, and human welfare. Research oriented towards a better understanding of the joint effects of land use and climate change over the provision of specific ecosystem services is therefore essential to safeguard such services. Here we propose a methodological framework, which integrates species distribution forecasts and graph theory to identify key conservation areas, which if protected or restored could improve habitat connectivity and safeguard ecosystem services. We applied the proposed framework to the provision of pollination services by a tropical stingless bee (*Melipona quadrifasciata*), a key pollinator of native flora from the Brazilian Atlantic Forest and important agricultural crops. Based on the current distribution of this bee and that of the plant species used to feed and nest, we projected the joint distribution of bees and plants in the future, considering a moderate climate change scenario (following IPPC). We then used this information, the bee’s flight range, and the current mapping of Atlantic Forest remnants to infer habitat suitability and quantify local and regional habitat connectivity for 2030, 2050 and 2080. Our results revealed north to south and coastal to inland shifts in the pollinator distribution during the next 70 years. Current and future connectivity maps unraveled the most important corridors, which if protected or restored, could facilitate the dispersal and establishment of bees during distribution shifts. Our results also suggest that coffee plantations from eastern São Paulo and southern Minas Gerais States could suffer a pollinator deficit in the future, whereas pollination services seem to be secured in southern Brazil. Landowners and governmental agencies could use this information to implement new land use schemes. Overall, our proposed methodological framework could help design novel conservational and agricultural practices that can be crucial to conserve ecosystem services by buffering the joint effect of habitat configuration and climate change.

## Introduction

Understanding the joint effects of land use and climate change on biodiversity and the provision of ecosystem services has become a pressing need, highlighted by the United Nations’ Intergovernmental Platform on Biodiversity and Ecosystem Services (IPBES) [1,2]. Substantial efforts are thus needed to assess such combined effects, and then translate the generated knowledge into policies aiming to conserve or restore natural capital and ecosystem services [3–5].

*Mobile Agent-Based Ecosystem Services* (MABES) [6], or ecosystem services provided by mobile agents, are increasingly threatened by the human-mediated modification of natural habitats as well as by climate change [7–10]. MABES declines could have important negative ecological and economic consequences, because they could hinder the maintenance of wild plant diversity [11], narrow ecosystem stability [12], reduce crop production [13–15], threaten water availability [16] and affect human health [17,18], decreasing human welfare.

Species range shifts due to climate change have already been extensively reported, and comprise a wide range of taxa and regions. Previous studies have suggested some patterns, such as distribution shifts towards the poles [19,20] and higher elevation expansion ranges [21], but more complex and sometimes unexpected distributional shifts are also common [22]. These shifts highlight the importance of more studies assessing multiple species and sites.

On the other hand, increasing habitat connectivity is commonly quoted as an important management practice to facilitate species relocation to more suitable habitats [23]. Although it is true that habitat connectivity and landscape fragmentation have been traditionally discussed in an island-biogeography or metapopulation theory framework, recent contributions from the field of landscape ecology have highlighted the importance of expanding this approach to incorporate other landscape characteristics, including landscape composition and configuration and matrix permeability [24]. So we adopted a landscape ecology approach that goes beyond the island biogeography by using species dispersal characteristics, graph theory and also species distribution models to analyze the landscape characteristics, identify areas with higher potential to shelter populations in current and future climatic conditions and propose management actions to promote species conservation. Higher connectivity can contribute for increasing the resilience of population under multiple stressors [25], enhancing also gene flow [26], colonization rates [27], and decreasing extinction risks [28]. However, most connectivity analyses are still focused on current distributions that will likely be insufficient for protecting species whose distributions are changing [29,30]. Static geographic distribution models might not adequately account for species’ ability to disperse when seeking suitable areas [31]. Therefore, more complex integrative climate models, joining habitat fragmentation and species dispersal capabilities have to be considered in order to assess whether a focal species is able to reach new suitable areas when facing climate changes.

Combining distribution models with dispersal analysis was already proposed to identify the spatial cohesion of landscapes at a large spatial scale, based on the probability that an individual leaving one patch would arrive in another patch when dispersing to new suitable habitats [32]. Landscape genetic analyses were also used to forecast the impact of climate change on habitat connectivity in a North American marten [33]. Moreover, grid cell values of current and future climatic suitability, obtained through species distribution modeling, were used to rank top-priority areas for amphibian species, aiming to suggest priority areas for conservation [34,35]. However, to our knowledge, no study has yet provided an integrated approach that allows a systematic identification of the best areas for conservation and restoration considering dispersal capabilities, habitat connectivity and changes in climate along time using a MABES as study model.

Here we propose a methodological framework to help planners identify key locations that are important now and that will be important in future scenarios to preserve MABES. We applied our proposed framework to the provision of pollination services by a tropical stingless bee (*Melipona quadrifasciata*), native to the Brazilian Atlantic Forest. Pollination services are among the ecosystem services most impacted by habitat loss and fragmentation [36–39]. Key pollinators, such as wild bees, have proven susceptible to the degradation of natural habitats, as several studies have shown that bee abundance and richness are negatively affected by habitat loss and fragmentation [40–42]. Likewise, climate change was suggested as causing reduction in bee pollinators [43–45], affecting interaction patterns [46], plant-pollinator phenology [47–49] and spatial distribution [50,51].

We first modeled shifts in bee pollinator range driven by climate change. Then, we used the resulting distribution to perform habitat connectivity analysis based in graph theory. By so doing, we were able to identify the most relevant areas where pollination services provided by this bee will change more intensively during climate change. We discuss the advantages and caveats of our approach, and suggest how it can be best applied to other MABES.

## Methodological Framework

### Study species and area

Our study model is native to Brazilian Atlantic Forest, which is known to be a biodiversity hotspot threatened by habitat loss and fragmentation [52–54]. Moreover climate change represents a serious menace to survival of several species native to this ecosystem [55–59]. Increasing connectivity among Atlantic Forest remnants can thus be particularly important to allow species relocate to suitable habitats. Recent actions have already been taken to protect and restore the Atlantic Forest in order to ensure connectivity [60,61], but to date none of these actions considered the joint effects of land use and climate change.

*Melipona quadrifasciata* is an important pollinator of many native plant species of the Atlantic Forest [62,63], as well as economically important agricultural crops, including coffee, pumpkin and tomato [64–66]. Although *M*. *quadrifasciata* can be found foraging on agricultural areas, it is usually associated to preserved forest fragments since it depends on large trees for nesting [67]. It has a well-known geographic distribution, ranging from north to south of Brazil [68]. Although the existence of two subspecies has been suggested [68], we considered the full species distribution in this study. Mutualistic interactions with native plants that provide the bees with sources of pollen, nectar and nesting sites have been relatively well documented [62,63,69,70].

### Framework pipeline

Our framework consists of three main steps: A) Modeling species’ potential distribution considering current and future climatic conditions; B) Combining species’ potential distribution with the native forest remnant map in order to use the habitat configuration to estimate habitat availability in current and future distribution areas; and C) Identifying main areas of species distribution variation (where pollination provided by this bee will increase or decrease) and key areas for conservation and restoration in order to allow the bee’s relocation to suitable habitats.

#### A) Habitat suitability

**Fig 1:**
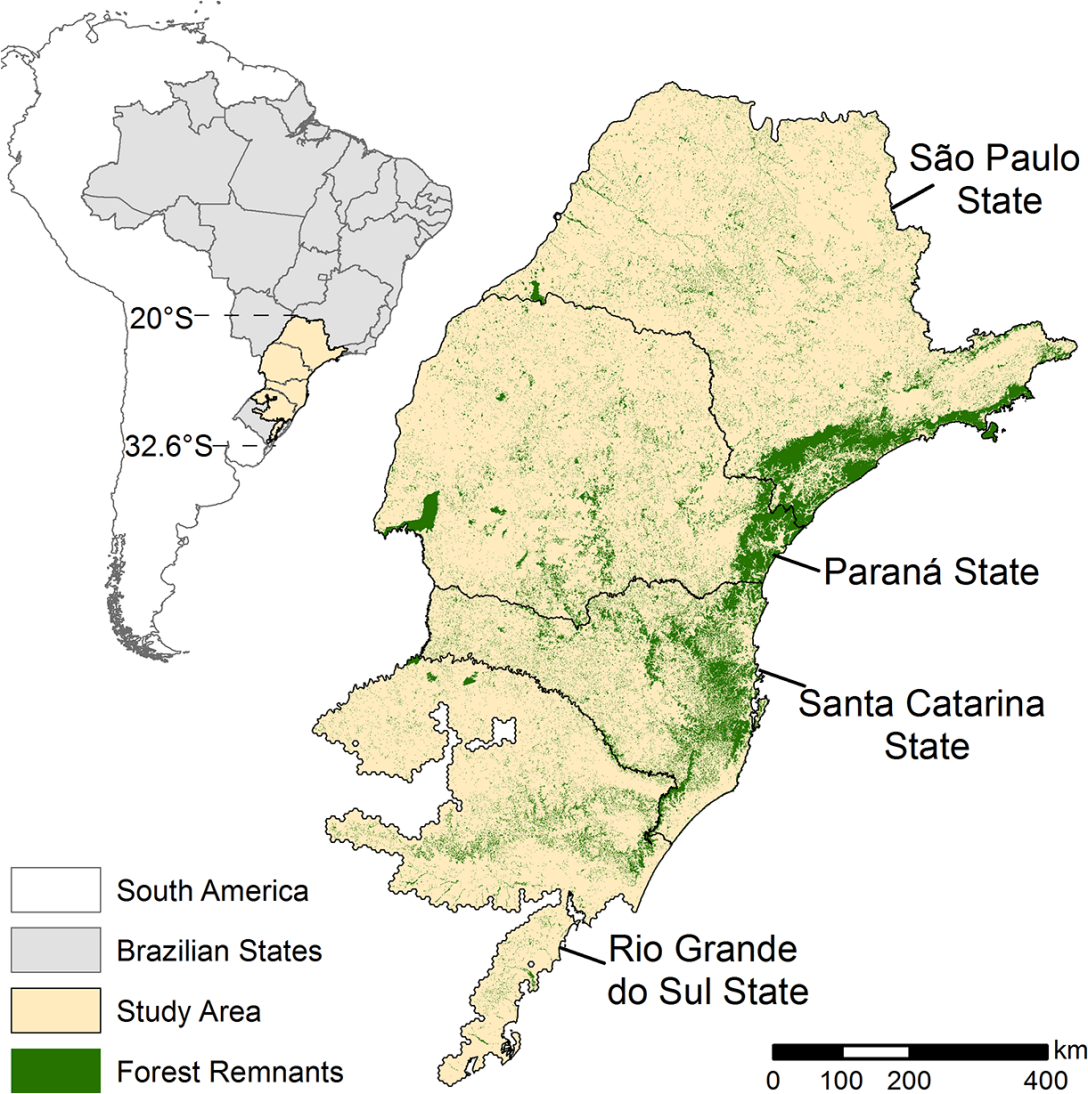
Forest remnants in the studied area, southern region of Brazil (forest remnants map was provided by SOS Mata Atlantica and Instituto Nacional de Pesquisas Espaciais 2008).

**Fig 2:**
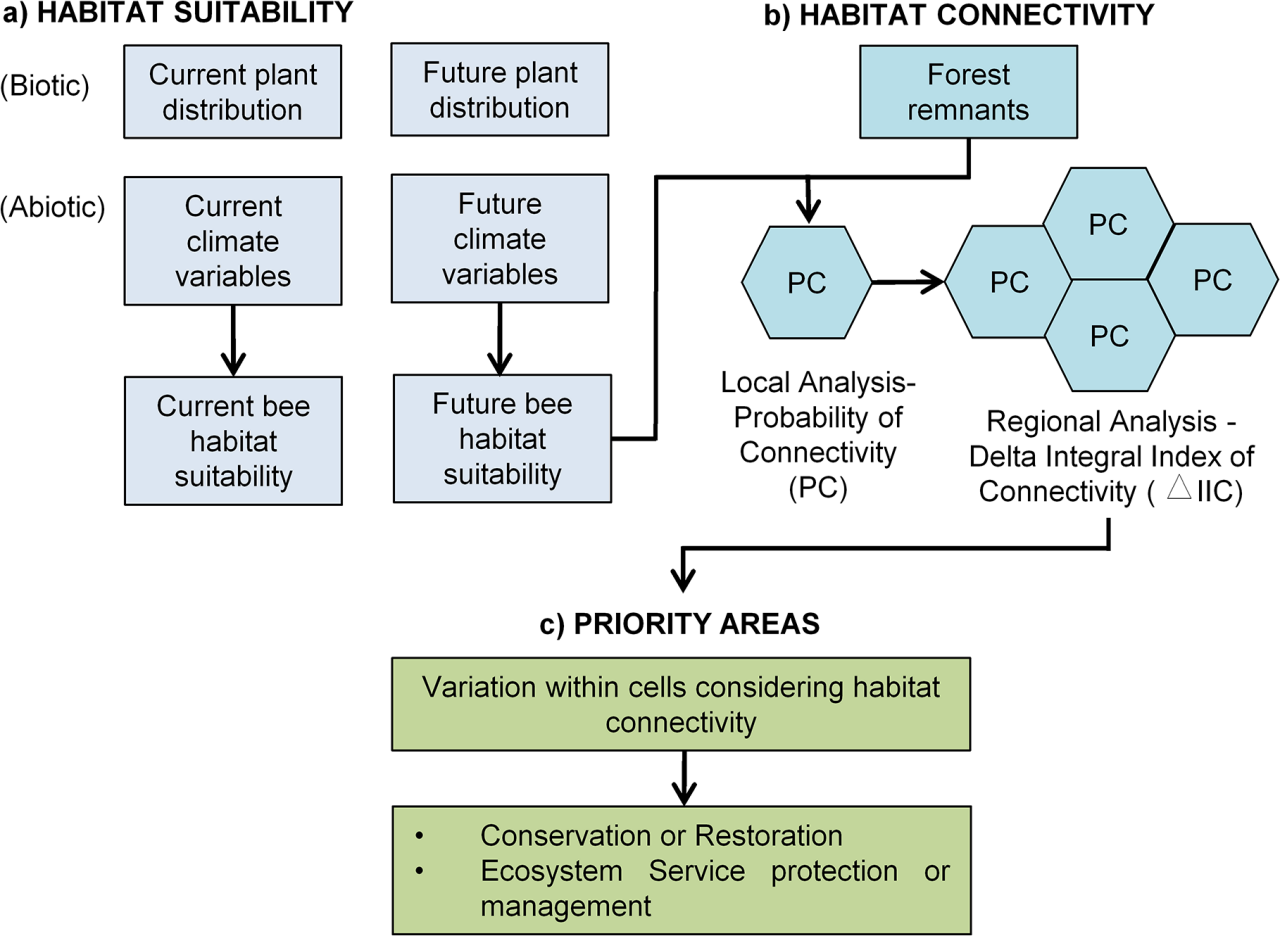
Methodology workflow: (a) Distribution modeling of *Melipona quadrifasciata* species included potential distribution of plants used to nest and to collect pollen and nectar (biotic factors) and climatic variables (abiotic factors). This modeling resulted in one present day and three future models (2030, 2050, 2080) of habitat suitability for the bee species (see item A on Material and Methods section and Fig 3A on Results section). (b) Local scale analyses estimated the habitat connectivity in each focal landscape (FL, hexagonal cells) through the Probability of Connectivity Index (PC). The PC was based on the bee dispersal capability and also on forest remnant areas that were weighted by habitat suitability obtained with the species distribution modeling (previous step). On regional scale, the importance of each FL to the potential bee flux through the study area was measured based on removal experiments, which estimate the contribution of each FL in changes in the Integral Index of Connectivity (ΔIIC) (item B on Material and Methods section and Fig 3B on Results section). (c) The determination of priority areas for conservation and restoration and for ecosystem services protection and management was based on temporal changes in FL regional importance (item C on Material and Methods section and Fig 3C on Results section).

To estimate the impact of climate change on the geographic distribution of the focal bee on the study area (Fig 1), we performed species distribution modeling using reported species occurrence locations and different environmental variables that can potentially affect species occurrence [71] (Fig 2, item a). Distribution models are considered useful to depict habitat suitability for species since they reflect the species responses to environmental features [72,73]. As distribution models are not usually adequate to be used on fragmented landscapes, we included a connectivity analyzes based on the forest remnant map. This aims to identify potential distribution areas based on climatic conditions and also, on habitat configuration.

Occurrence locations of *M*. *quadrifasciata* were retrieved from a Brazilian diversity database that provides information of museums and entomological collections (http://www.splink.org.br/) and were complemented by data presented in [68]. Environmental variables were composed of climatic variables obtained from Worldclim website [74] with a resolution of 30 arc-seconds (~1 km). From an original set of 20 variables, we calculated the eight least correlated ones for the study area (following [75]), which are: Annual Mean Temperature; Isothermality; Max Temperature of Warmest Month; Min Temperature of Coldest Month; Mean Temperature of Driest Quarter; Precipitation of Wettest Month; Precipitation of Wettest Quarter; and Precipitation of Coldest Quarter.

Since *M*. *quadrifasciata* exhibits important mutualistic interaction with some tree plant species, we also included three biotic environmental variables based on the potential distribution of trees that provide nesting, pollen, and nectar resources (following [76]). Occurrence data for plants was retrieved from the same data source. We considered *Tibouchina granulosa* as nest source [77]; *Solanum inaequale*, *Bathysa meridionalis* and *Machaerium nyctitans* as nectar sources [62,63]; and *Sclerolobium denudatum*, *Cupania oblongifolia* and *Solanum granuloso-leprosum* as pollen sources [62,69,70]. We modeled each plant species separately to obtain their potential distribution. After this, we normalized all models and summed those models belonging to nectar sources (three species) and those belonging to pollen sources (three species), thus resulting in three biotic variables (potential distribution of nectar sources, pollen sources and nesting site). Following this procedure, we used a total of 11 environmental variables (eight climatic and three biotic variables) to forecast bee habitat suitability in current climate conditions.

In order to analyze future scenarios we used the projections made by the Canadian Centre for Climate Modelling and Analysis (CCCMA) for a moderate future climate change scenario (A1B; [78]. The same eight climatic variables from three periods, 2030, 2050 and 2080, were obtained from the International Center for Tropical Agriculture website [79]. As future biotic variables, we used the future potential distribution of the same resources plant species already mentioned. We used the Maxent algorithm (Maximum Entropy) [80] that is adequate to presence-only data [81]. We estimated models accuracy using ROC-AUC (area under receiver-operating curve) where values near 1.0 indicate good results [82].

#### B) Habitat connectivity

All connectivity analyses were based in graph theory, which is a simplistic but robust approach to incorporate functional attributes in habitat connectivity analyses [83] and is considered a measure of habitat availability [84]. In the representation of a landscape as a graph, each patch is usually considered a graph node. Each node has its own attributes (e.g. related to the size, quality or amount of habitat) and can be connected to other nodes by links, which represent functional connections in the landscape.

As *M*. *quadrifasciata* is highly dependent on native remnant vegetation [63], we consider only two land cover classes (forested and non-forested areas), provided by the Atlantic Forest remnant vegetation map [85]. The study area was firstly divided into several equal size hexagonal cells with 5,000 ha which were considered our focal landscapes (FL) and the connectivity inside each FL was calculated using the index Probability of Connectivity (PC; [86]; Fig 2, item b, Eq 1). The PC is considered a robust index to measure connectivity and is calculated by the following formula [86]:

$$PC=\frac{\sum_{i=1}^{n}\sum_{j=1}^{n}a_{i}a_{j}p_{ij}^{*}}{A_{L}^{2}},$$ Eq 1

where *n* is the number of nodes in the landscape; a_i_ and a_j_ are the attributes of nodes *i* and *j*; p^*^_ij_ is the probability of connection between nodes *i* and *j*; and *A*_*L*_ is the landscape area.

To incorporate species habitat suitability in the PC, we used as node’s attribute the remnant vegetation area weighted by the habitat suitability values generated by the distribution modeling. To do this, we first resampled the raster of the bee potential distribution model to the same spatial resolution of the forest cover map (50 m). Then, the habitat suitability values of all pixels that intersect each forest patch were summed to obtain the node’s attribute (i.e. the attribute of each forest patch). Thus, a forest patch that presented both high habitat suitability and large area will have a higher value of node attribute when compared to a small patch located in a low habitat suitability region. Finally, this area weighted by the distribution model was used as node’s attributes in the calculation of the PC. The probability of connectivity between two patches was estimated based on a negative exponential function (used by the software Conefor Sensinode 2.5.8; [87]). This function was parameterized considering species flight range reported by [88], where we considered a 10% probability of functional connection between patches with 2 km distant from each other.

Although all analyzed FL had the same area, the maximum sum of attributes of each landscape was not the same due to the weighted criteria adopted, described in the previous paragraph. Thus, we only used the numerator of the PC to give higher importance for landscapes with higher habitat connectivity and higher habitat suitability according to the species distribution models.

Previous studies have shown that besides patch area and isolation, other landscape features such as patch shape, patch perimeter, matrix characteristics and habitat quality can influence patch invertebrates density and migration through the landscape [89,90]. However, the effect of patch topology can exhibit a remarkable variation, depending on organism size, life history characteristics and foraging behavior [91,92]. As we do not have detailed data about the effects of patch topology on migration rates of our studied species, we only considered the dispersal distance available from the literature to estimate dispersal probabilities

In a second step, we conducted a regional scale analysis to estimate the importance of each FL for the potential bee flux through the whole study area. In this regional scale analysis, the entire study area was then considered a graph: the FL was the graph’s nodes and the nodes’ attributes were the PC numerator calculated in the previous step (see more details on this procedure in [93]). In this regional scale analysis we calculated the Integral Index of Connectivity (IIC) [94] which is a binary version of the PC. Only the adjacent nodes were considered to be functionally connected because the hexagons edges length is 4.3 km which is higher than the bee’s dispersal capability. To identify the importance of each FL for *M*. *quadrifasciata* dispersal, IIC was calculated for the study area and then nodes were subsequently removed using the program Conefor Sensinode 2.5.8 [87]. After each FL was removed, the IIC of the study area was recalculated. The variation of the IIC (ΔIIC) after the removal of each FL was considered to be the importance of each FL for regional dispersal. All connectivity analyzes were conducted considering the current and future distribution models of the bee, resulting in four maps (current, 2030, 2050, 2080; Fig 2, item b). We conducted a sensitivity analysis to check the influence of variations on dispersal distances in the regional importance of each landscape, and our findings suggested that these variations do not affect the final results (see S1 Fig for details).

#### C) Determination of changes in the provision of pollination services and identification of priority areas for conservation and restoration

To define priority areas for conservation and restoration, as well, to highlight changes in the ecosystem services delivery, we considered temporal changes in habitat connectivity measured at regional spatial scales (Fig 2, item c), here represented by the changes in ΔIIC values. We considered that these changes would reflect a potential change in the provision of ecosystem services since it incorporates both changes in environmental suitability and the landscape structure that will modulate the movement of bees inside and between focal landscapes.

The variation in ΔIIC was estimated to all FL of the study area, considering three periods of time: 1) from present day to 2030; 2) from 2030 to 2050; 3) from 2050 to 2080. This allowed us to see whether connectivity increased, decreased or showed no difference through time. We subtracted values of future connectivity from the previous one, which means, we calculated the changes in ΔIIC for each FL considering the following calculations: 1) 2030—present day; 2) 2050–2030; and 3) 2080 – 2050. To also obtain an overall trend of the shifts between the whole period (present day to 2080), we subtracted the values of connectivity obtained per each cell for 2080 model from the values obtained per each cell for the present day model. Once the model did not consider changes in habitat cover, areas where connectivity is increasing represent areas where habitat suitability is increasing due to direct or indirect (e.g., through changes in plant species distribution) effects of climate changes.

Consequently, priority areas for conservation and restoration should consider not only areas where connectivity will increase in the future, but also those where connectivity will be maintained high (and that can act as source of individuals for future suitable areas). On the other hand, changes in connectivity also reflect potential changes in the provision of ecosystem services, and should be useful to consider this in agricultural management actions in order to mitigate the loss of services or to take advantage of its increase.

**Fig 3:**
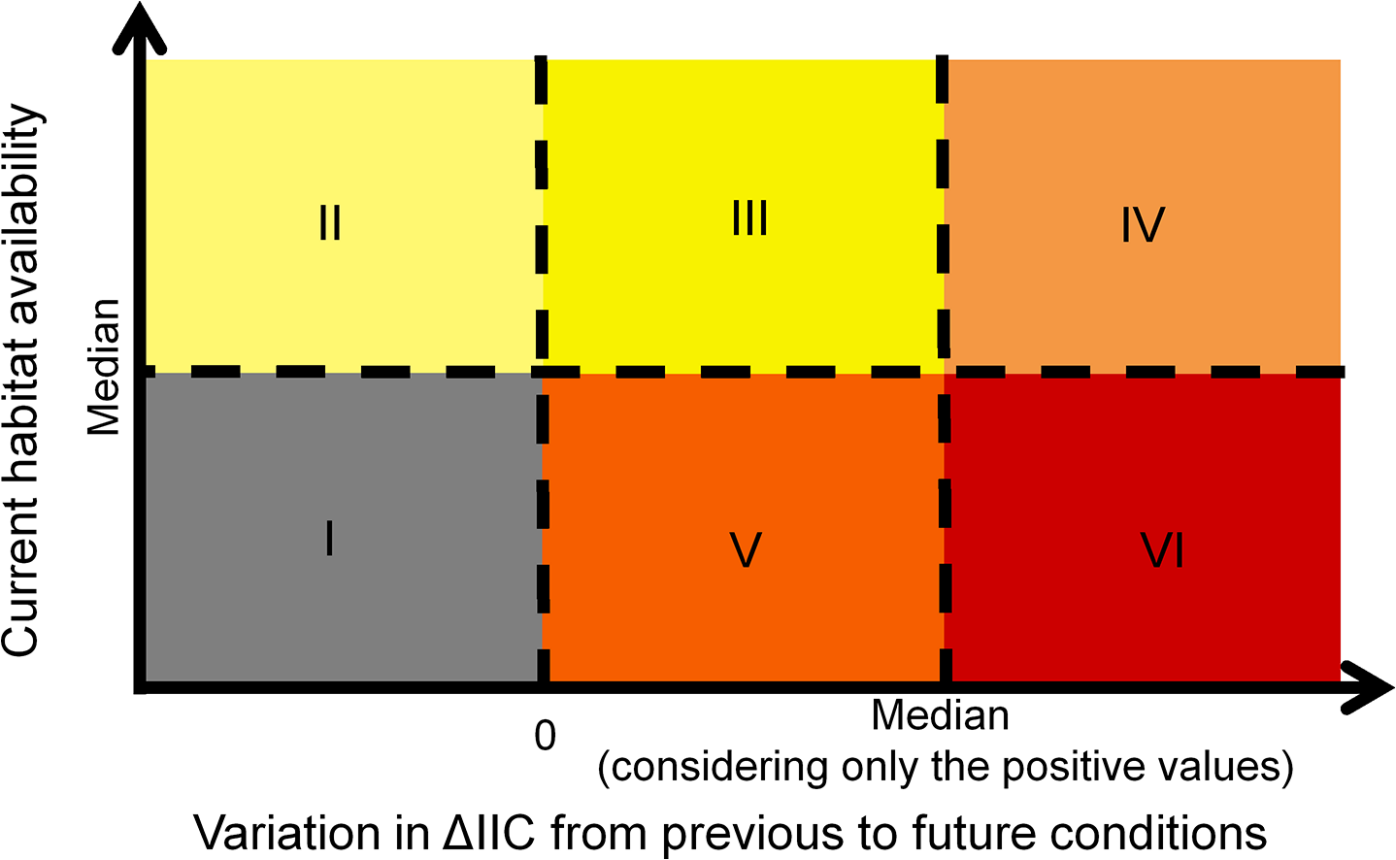
Criteria used to define ideal conservation and restoration strategies, considering initial habitat availability and the variation in the importance of each focal landscape due to changes in future environmental conditions (variations in ΔIIC). The six suggested strategies and their justifications are: I- no action: current conditions are below median and will decrease in future scenarios; II- short-term conservation: current climatic conditions and connectivity are favorable, but conditions will decrease. Regions may act as species source for migration; III- low priority for restoration actions and long-term conservation: current climatic conditions and connectivity are favorable and conditions will increase. Restoration may increase habitat availability; IV- Long-term conservation actions: current and future conditions are favorable. Regions may maintain species during climatic change; V- Intermediate priority for restoration: current conditions are bellow median but will increase in future scenarios. Restoration actions would increase habitat availability and maximize species conservation potential; VI- High priority for restoration: current conditions are below median, but will increase in future scenarios. Restoration actions would increase habitat availability and have maximum potential of benefits for species conservation. See Material and Methods section for the full description of the criteria used to define conservation or restoration strategies.

Based on habitat availability and future climatic conditions, we suggested conservation strategies based on the current and future value of different areas. We adopted simple criteria to decide whether and when a given area should be the focus of conservation or restoration strategies. Areas were divided in 6 classes (Fig 3): **I) No action** are indicated to landscapes that present the worst current conditions with decreasing adequate conditions in the future. Restoration or conservation actions may not obtain good results in these areas. **II) Short-term conservation actions** are indicated to landscapes that present habitat availability above the median value in the first period analyzed (measured through the PC numerator index) and that presented a decrease in ΔIIC in future scenario. These landscapes can be considered sources of individuals in current conditions, but would not be adequate for long-time species persistence. **III) Low priority for restoration and long-term conservation actions** are indicated to landscapes that present habitat availability above the median value in the first period analyzed, and will also present a small increase (below the median) in ΔIIC in future scenario. In these landscapes, restoration actions may increase habitat availability and maximize species persistence, but current and future conditions are already good, and thus should be conserved in long-term. **IV) Long-term conservation actions** are indicated to landscapes that present current habitat availability and increase in ΔIIC above the median. These areas can be considered adequate for species persistence in current and future conditions. **V) Intermediate priority for restoration actions** are indicated to landscapes that present current habitat availability below the median, but will present an increase in ΔIIC in future scenarios. Restoration in these landscapes might increase conditions for species persistence. **VI) High priority for restoration actions** are indicated to landscapes that present current habitat availability below the median, but will present higher values of ΔIIC improvement in future scenarios. Restoration actions to improving landscape connectivity in high priority landscapes will have high potential for species conservation due to high climatic suitability, and thus, will increase the chances of establishment of new populations that will migrate from areas with decreasing environmental conditions. In our example, we calculated the median value of the variation in ΔIIC considering only those values that were positive to make sure that restoration strategies would be conducted in areas that will present an increase in environmental conditions in futures scenarios. However, the thresholds used to define the classes can be easily adapted to the available budget and objectives of conservation and restoration, increasing or reducing the amount of priority areas.

## Results

**Fig 4:**
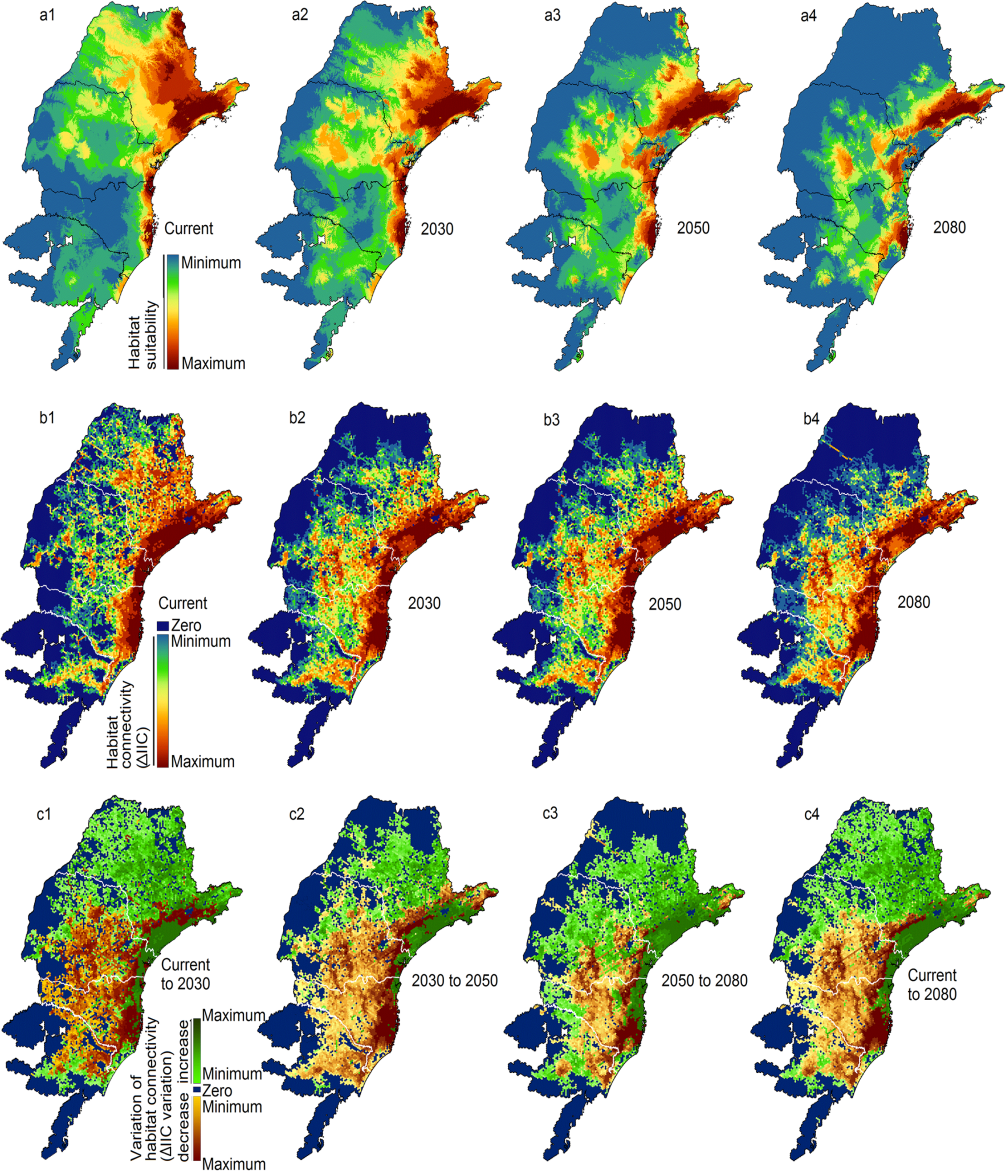
(a) Bee habitat suitability according to species distribution model outputs for (a1) current conditions and for (a2) 2030, (a3) 2050, and (a4) 2080 scenarios of climate change. Modeling was executed using climatic variables (abiotic factors) and mutualistic plant species (biotic factors) (see item A on Material and Methods section). (b) Habitat connectivity of each focal landscape (FL) represented by the variation of Integral Index of Connectivity (ΔIIC) through the study area, for (b1) current conditions and for (b2) 2030, (b3) 2050, and (b4) 2080 scenarios. Since the importance of each FL is measured by ΔIIC, the highest the ΔIIC the highest the FL importance (item B on Material and Methods section). (c) Changes in habitat connectivity represented by the variation in ΔIIC considering two climatic subsequent periods: (c1) current to 2030; (c2) 2030 to 2050; (c3) 2050 to 2080; (c4) current to 2080.

Distribution models presented good accuracy (AUC > 0.9), and they suggest a reduction in habitat suitability during the analyzed period of time, as well as a shift from north to south (Fig 4A) (details can be found on S2 Fig). More specifically, there will be a potential decrease in habitat suitability on the current northern distribution range (São Paulo State mainly), resulting in a distribution restricted to the eastern coastal line. However, on the central and south extents of the current distribution area, models indicate a potential increase in suitability, especially in inland areas.

Habitat connectivity analyses of suitable areas for bees reveal highly connected areas on the coastal line over the whole period of time (darker red on Fig 4B). Only a small coastal area in southern São Paulo will present a potential decrease in importance for regional connectivity. Inland areas and almost all coastal areas on the south constitute important regions where bees will be able to find suitable habitats and migration corridors.

**Fig 5:**
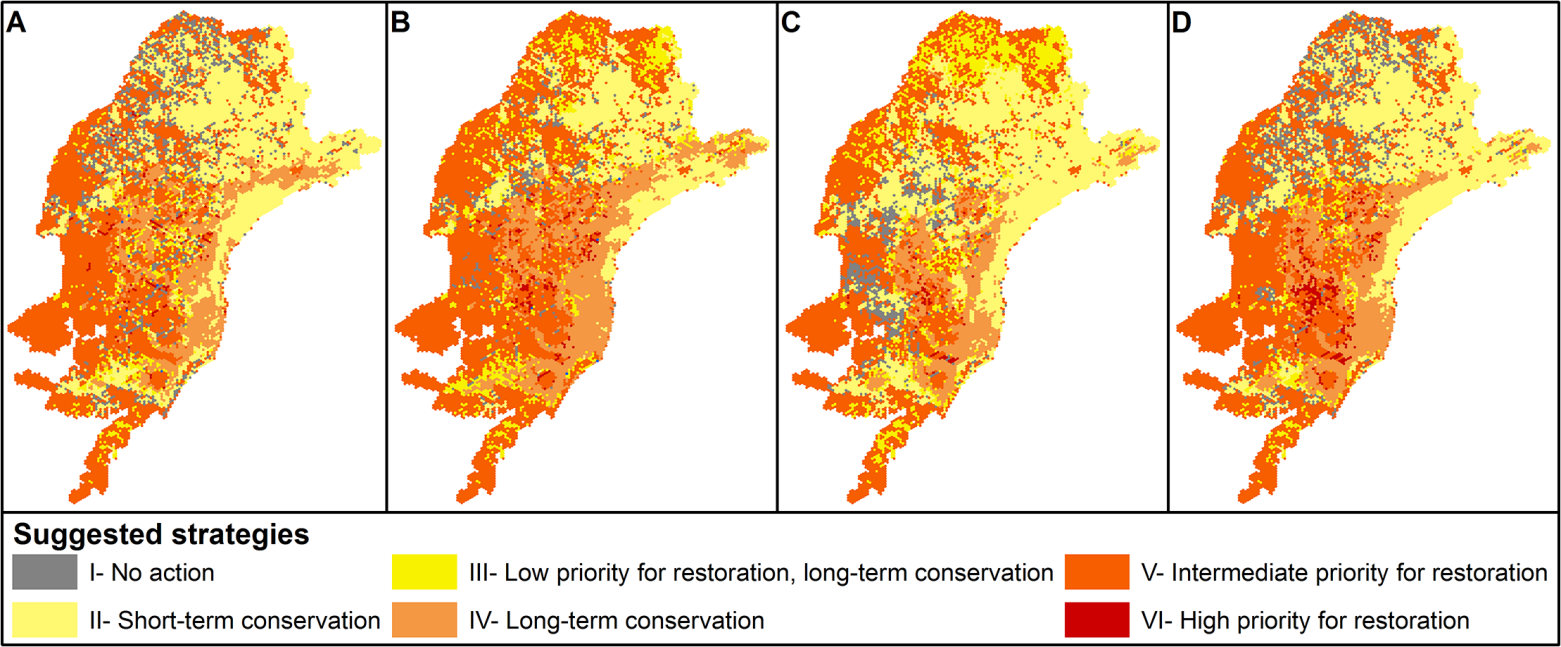
Suggested conservation/restoration strategies for the focal landscapes based on previous habitat availability and changes in future climatic conditions, considering the two subsequent periods: (A) current to 2030, (B) 2030 to 2050, (C) 2050 to 2080, and (D) current to 2080. See Fig 3 and the Material and Methods section for a full description of the criteria used to define these strategies.

Most focal landscapes show higher variation in regional connectivity, measured by changes in ΔIIC when comparing the current conditions to 2030 (darker red and darker green on Fig 4C). The comparison of regional connectivity between 2030–2050, 2050–2080 and current to 2080 periods show that most cells present lower variations (light green and light yellow on Fig 4C) and the higher variations are more aggregated when compared to changes between current and 2030 (darker colors on Fig 4C). Southern areas can be considered as priority areas for restoration actions since most of them present an increase in regional connectivity during the analyzed period (dark red on Fig 4C), but also present low current habitat availability (Fig 5A). The northern areas will present a decrease (dark green on Fig 4C) in regional importance, but they present higher values of importance in current conditions, so they should be considered important areas for short-term conservation (Fig 5) due to their potential as individual sources and possible migration routes. The coastal region presents a high number of focal landscapes with high regional importance and with increasing importance in future scenarios (Fig 4). The high values of current and future conditions suggest that the coastal landscapes should be considered high priority for long-term conservation actions (Fig 5) due to its importance for maintaining the species in current and future conditions, and also to act as sources of individuals to colonize new suitable habitats in long-term.

## Discussion

The proposed methodological framework allows to link species connectivity modeling with species distribution modeling, resulting in an integration of habitat configuration and climate change effects on a species distribution. It has implication on conservation and restoration actions, as well on ecosystem services delivery, being useful to public policies and to agricultural practitioners, helping them to deal with changing environments.

### Conservation and restoration implications

Climate forecast showed that the future distribution of our pollinator species will contract on its northern range but will expand to southern coastal and inland regions of the Atlantic Forest. Southern areas were identified as priority areas for restoration and conservation since most of them will present suitable habitats during the future analyzed period, while northern areas will overall present a severe suitability decrease. This result becomes clearer when distinguishing the areas that are more important to maintain current connectivity from the areas that are more important to maintain future connectivity. Whereas southern areas will be important in the future, northern areas are essential to maintain current connectivity.

A key outcome is that our analyses reveal different conservation needs for different timeframes and areas (Fig 5 items a-d). Small variations on the importance of each focal landscape when considering minor time intervals allowed us to identify possible ecological corridors to facilitate species displacement due to climatic changes in a near future. The high variation in focal landscape importance when analyzing only one large time interval (current to 2080) might difficult the identification of possible dispersion routes during the initial phases of climate change. Thus, adopting multiple short time intervals to design land use plans is essential to identify the potential biological corridors and create a long-term conservation work plan. Current conservation efforts should focus in protecting northern areas mainly on São Paulo State, whereas long-time conservation programs should invest more in southern areas of Parana and Santa Catarina states. Planners can thus identify locations that are important for species maintenance now and that will be important considering different time steps of future scenarios of climate change. It is essential to consider the condition shifts through time, in order to maximize ecological benefits and reduce restoration costs [95,96].

Due to high costs of conservation/restoration practices, it is essential to identify areas with high current and future suitability and connectivity, such as the coastal areas in our studied region (Figs. 4 and 5) to guarantee the highest return when implementing such practices. Protection of sites may be more cost-effective in the species’ geographic distribution areas where habitat is predicted to remain suitable over time. Since forest restoration actions take ca. 10–20 years to show results, it seems to be more adequate to consider steps of ca. 20 years when planning restoration actions on future suitable areas to guarantee connectivity. Those areas could then provide suitable habitats for source populations, from which the species might expand if or when conditions allow it [32]. Actions considering distribution modeling or habitat connectivity independently could result in an inadequate prioritization of areas, potentially conducting to ineffective land management.

Different actions can be used to ensure connectivity in the identified priority areas. One possible action consists of protecting current forest remnants that are well preserved nowadays and that will be important in the future. Other possible action consists of developing functional corridors between priority areas, either by creating new protected areas or by providing areas already protected with stepping stones of suitable habitats. Since creating new patches of habitats is costly, it could also be useful to enrich the existent ones. For pollinators, patches can be provisioned with plant species known to be important for them. This would also benefit other wild bee species (e.g., 14 other *Melipona* species are reported in our study area according to specieslink data provider). It is also important to adopt a local-scale spatially explicit approach to identify the ideal areas to create new patches or enrich existing patches [97], in order to optimize restoration efforts and maximize the habitat availability for our focal pollinator species.

### Ecosystem services implications

Our findings have also important implication for protecting pollination services. Our focal bee is an important pollinator of agricultural crops, as many other native stingless bees [98,99]. The predicted climate shifts in the distribution of this species are thus expected to result in a potential deficit of pollination in some areas and a subsequent fall in crop yields. Traditional coffee plantation from the interior of the states of São Paulo and Minas Gerais, could be particularly affected [100–103]. In order to safeguard pollination services and avoid future pollinator deficits, it is important both to preserve the pollinators and to design agro-schemes according to the potential distribution of pollinators in future scenarios, considering their current distribution and probable dispersal routes.

Public management campaigns for pollination services protection can be also implemented, introducing relevant plants in areas surrounding croplands, since nest and flower resources are important in determining the spatial distribution of pollinators [42,104]. This was already proposed to help protect bee species that occur in dry forests on Northern Brazil [105] and to protect carpenter *Xylocopa* bees that pollinate passion fruit in the Tropical Savannas of Central Brazil [106,107]. The use of new crop varieties that are less dependent on *M*. *quadrifasciata* pollination could also help buffer the decrease in the availability in this pollinator. Alternatively, stingless beekeeping could be promoted as a sustainable development tool to help protect the bees and their pollination services [108].

Our results suggest that the distribution of our focal bee will expand to southern Brazil, thus bringing its pollination services to these regions. Pollinator-dependent crops in the study area include coffee (approximately 3 million of tons produced in 2012 according to agricultural database of The Brazilian Institute of Geography and Statistics—IBGE) (http://www.sidra.ibge.gov.br/), tomato (2 million of tons in 2012; IBGE) and pumpkin (270 thousands of tons; data available only for 2006; IBGE). Southern areas of Brazil were predicted to become more favorable to the cultivation of coffee due to future climate change [109], and in this case, the presence of our focal bee could bring additional benefits. Agricultural managers could use this information to mitigate or adapt to the predicted shifts, changing or diversifying their crops. The information provided by our framework can thus help to support agricultural management decisions.

### Modeling implications

Our proposed methodological framework can be adapted to study other organisms, and other ecosystem services provided by different species, as it only requires species occurrence data, basic information on habitat suitability and cartographic information that allows mapping suitable habitat and connectivity for that species. Possible caveats of distribution modeling usually includes the lack of knowledge about the full distribution of species that usually is surveyed on biased location, such as, areas where species was previously recorded, near research institutes or areas usually surveyed [110]. In spite of our species distribution has been recently fully described [68] we can consider that areas on the interior of Southern region (especially on Parana and Santa Catarina States) should be more surveyed to confirm the presence of this species. Additionally, other modeling techniques can also be used, including different algorithms, climate scenarios and ensemble forecasting, or interacting species.

Future studies could further refine our approach by including, for example, an estimate of future deforestation, modeling the distribution shifts in a larger number of species or testing different climate and land use changing scenarios. Particularly, we did not model future changes in land use and land cover in the Atlantic Forest, since Atlantic Forest has now under rigid protection and we can assume that there will be no more significant habitat loss in this biome. However, we included plant species in the modelling procedure aiming to obtain results that are more realistic about on species distribution changes, since previous studies have showed future potential shifts toward the south region for characteristic trees on this biome [55]. Despite its recent low net rates of forest loss [85], some regions of the Atlantic forest exhibit a dynamic pattern of deforestation and regeneration [111–113]. A higher future deforestation, although not probable, would further decrease habitat connectivity, thus changing the connectivity in some areas. Since restoration projects have being conducted on Atlantic Forest, our approach aims to contribute to the definition of priority areas using a methodological framework that considers both climate and habitat change.

Additional studies should also evaluate the relocation of species due to climate change. Indeed, we know little about large-scale responses of species to climate change across heterogeneous landscapes. Competitive pressure between species [114], mismatches in mutualistic interactions [50], and the influence of decreased habitat quality on foraging, nesting, or reproduction [115–117] may be important mechanisms determining whether species will be able or not to adapt to future climate space. The time required by bees to adapt to novel environments is a key parameter determining their ability to occupy new habitats but, unfortunately, there is no information on the literature about this. The only references available are the cases of invasive bee species, such as honeybees, that were able to colonize new habitats very quickly. For example, honeybees spread from Brazil to North America in 30 years [118]. But different species have varying life history characteristics, so we cannot speculate on the adaptation time of our study species.

### Concluding remarks

Overall, our work provides a clear methodological pathway to analyze how species will shift their distribution when facing climate change, and which are the main corridors that should be conserved or restored to help them to relocate to suitable habitats during a specified timeframe. It also illustrates how habitat connectivity analyses and species distribution modeling can be combined to assess the joint effects of habitat configuration and climate change on species distribution and on the delivery of ecosystem services provided by them. We believe our approach can be easily adapted to a wide range of species, helping support conservation and restoration programs.

## Supporting Information

S1 Fig Sensitivity analyses. Subset of the study area used to assess how the results are affected by changes in the bee’s dispersal distance (**Figure A**).
(DOCX)

S2 Fig Bee and plant occurrences and plant distribution models. Occurrences reported for *Melipona quadrifasciata* and the seven plant species used as biotic layers and the distributional model obtained for each plant. Models were based only on climatic variables (see Material and Methods for details) (**Figure B**).
(DOCX)
