# Supplementary material for: Safeguarding Ecosystem Services: A Methodological Framework to Buffer the Joint Effect of Habitat Configuration and Climate Change
Source: PLoS One. 2015 Jun 19;10(6):e0129225. doi: 10.1371/journal.pone.0129225 (PMC4475073; doi:10.1371/journal.pone.0129225)
Supplement: S1 Fig — Subset of the study area used to assess how the results are affected by changes in the bee’s dispersal distance (Figure A). (DOCX) [file pone.0129225.s001.docx]

**S1. Sensitivity analyses**

Sensitivity analyses were performed to assess how the results are affected by changes in the bee’s dispersal distance. To do so, we selected a subset of our study area (approximately 45% of the area, S1 Fig. item A) and repeated all analyses considering different dispersal distances. We calculated the numerator of the Probability of Connectivity (PC) considering current climatic conditions, and using four different combinations of dispersal distance and probability of connections associated to these distances:

1. dispersal distance = 2km and probability of connection = 0.1 (similar to the criteria adopted for the entire study area);
2. dispersal distance = 1 km and probability of connection = 0.1;
3. dispersal distance = 3 km and probability of connection = 0.1;
4. dispersal distance = 5 km and probability of connection = 0.5.

After the PC calculation for the different dispersal distance, we repeated the regional scale analysis to calculate the ΔIIC and determine the importance of each landscape for species migration through the study area. For this step, we repeated the criteria adopted in the study, considering only adjacent landscapes connected, since the size of each landscape (hexagon edge length = 4.3km) is much higher than all the simulated dispersal distances.

Finally, we used Kendall’s correlation tests to compare the results obtained with the different dispersal distances. All correlations were highly significant and showed high correlation coefficients (T>0.89, p<0.001; S1 items B, C, D), illustrating that our results are robust to changes in dispersal distance (S1 items E, F, G, H).


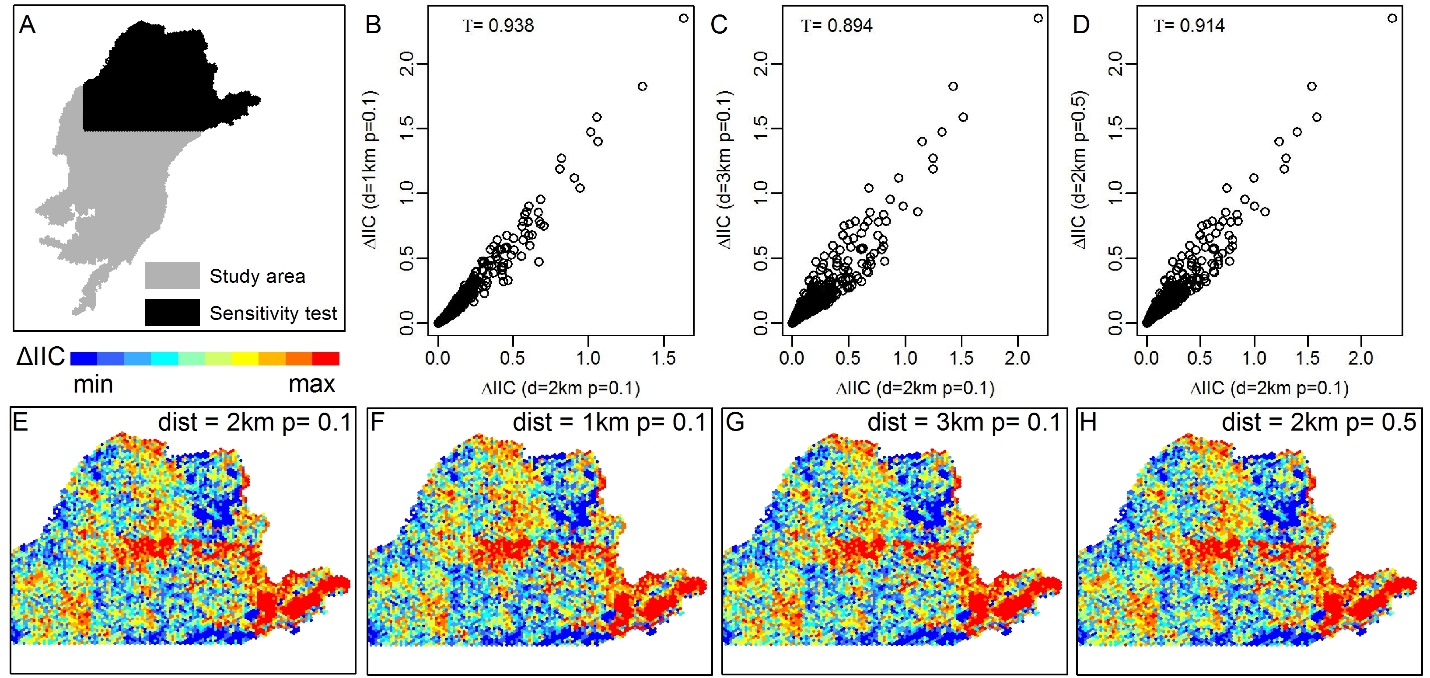


S1 Fig. Subset of the study area used to assess how the results are affected by changes in the bee’s dispersal distance.
