# Supplementary material for: Safeguarding Ecosystem Services: A Methodological Framework to Buffer the Joint Effect of Habitat Configuration and Climate Change
Source: PLoS One. 2015 Jun 19;10(6):e0129225. doi: 10.1371/journal.pone.0129225 (PMC4475073; doi:10.1371/journal.pone.0129225)
Supplement: S2 Fig — Occurrences reported for Melipona quadrifasciata and the seven plant species used as biotic layers and the distributional model obtained for each plant. Models were based only on climatic variables (see Material and Methods for details) (Figure B). (DOCX) [file pone.0129225.s002.docx]

**S2. Bee and plant occurrences and plant species distribution models**

S2 Fig. shows the occurrences for *Melipona quadrifasciata* and the seven plant species used as biotic layers on the modeling procedure. The models obtained for each plant species, based on climatic variables only, arte shown in the second column (see Material and Methods for details). Occurrence locations of *M. quadrifasciata* were retrieved from a Brazilian diversity database that provides information of museums and entomological collections (http://www.splink.org.br/) and were complemented by data presented in Batalha Filho et al. (2010). Occurrence data for plants was retrieved from the same data provider (http://www.splink.org.br/).

| 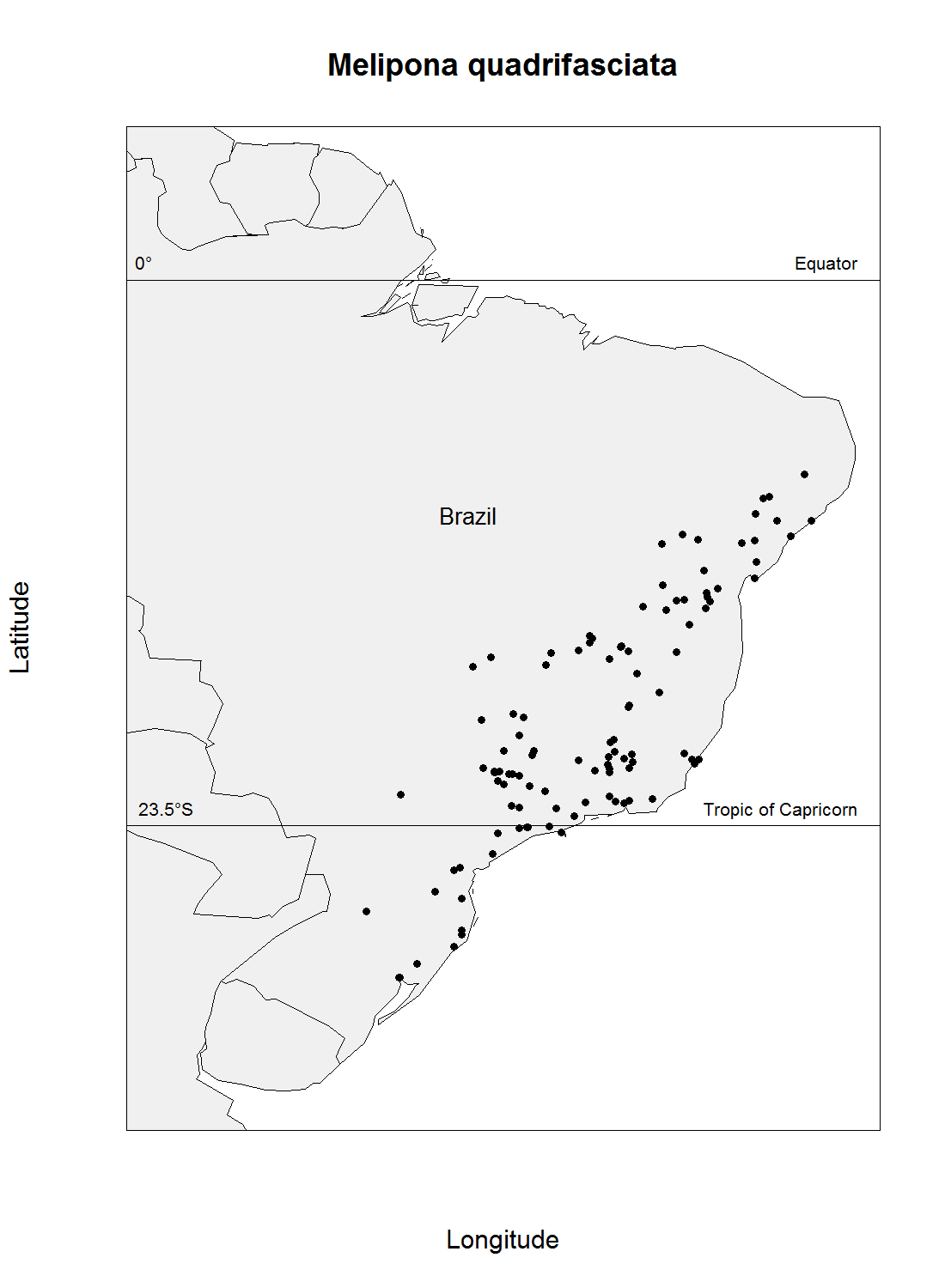 |  |  |
| --- | --- | --- |
| 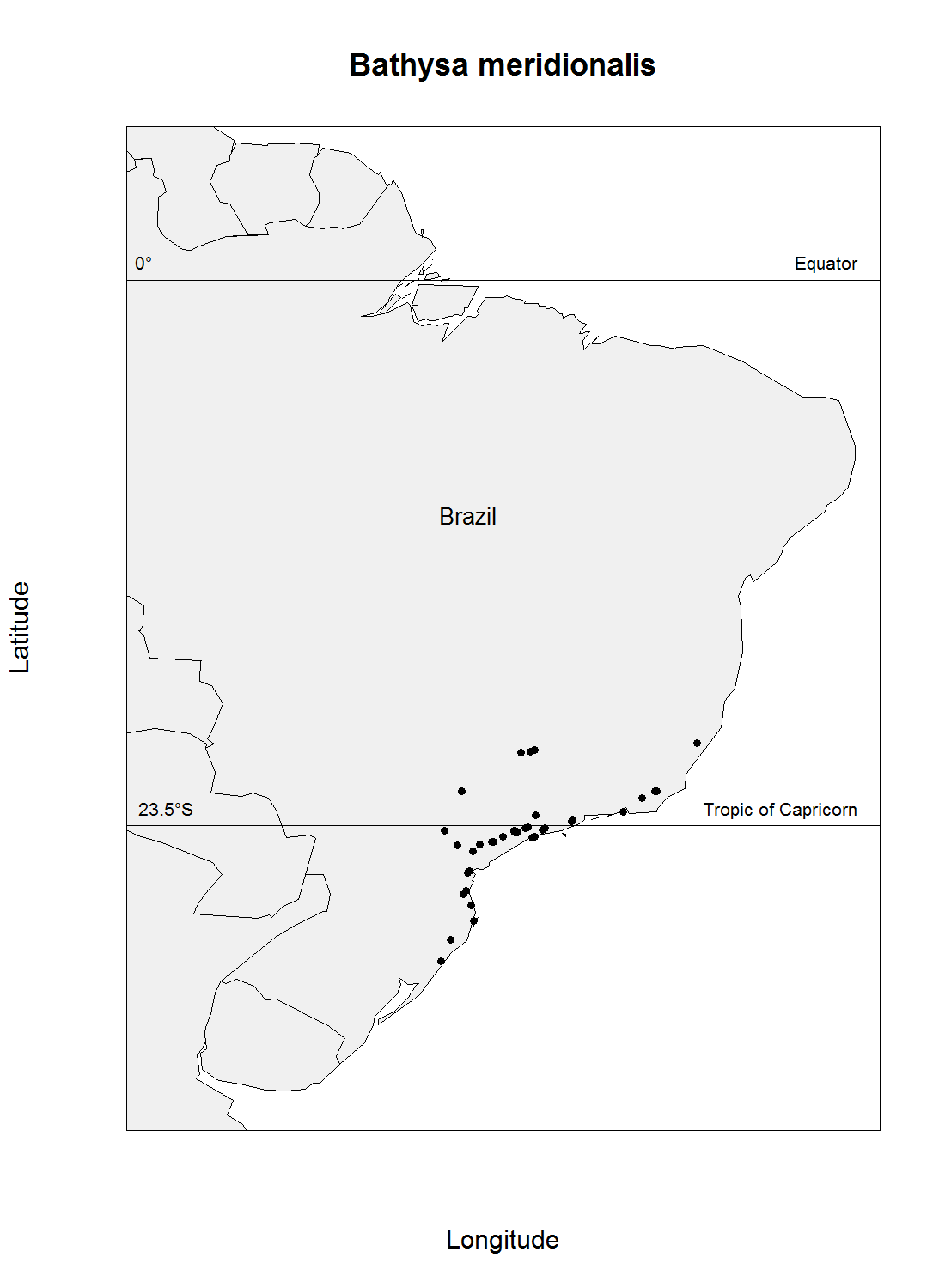 | 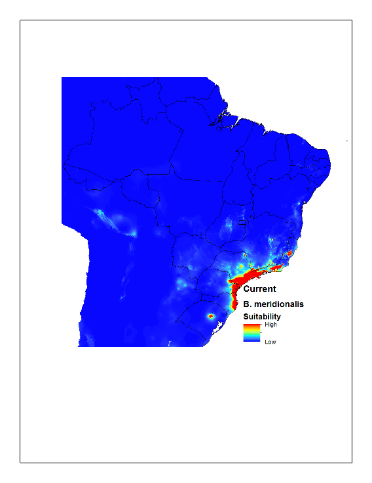 | 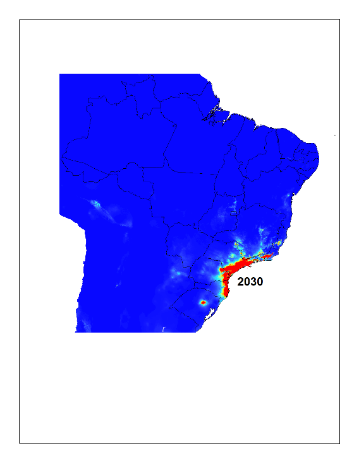 |
|  | 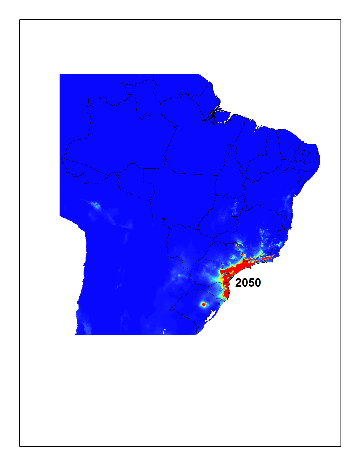 | 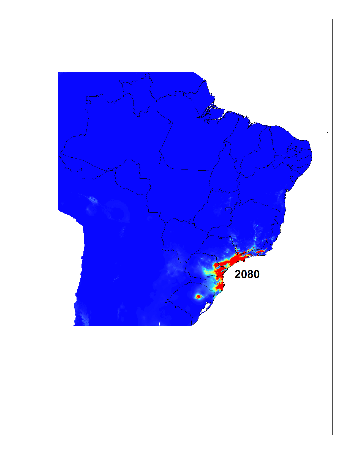 |

| 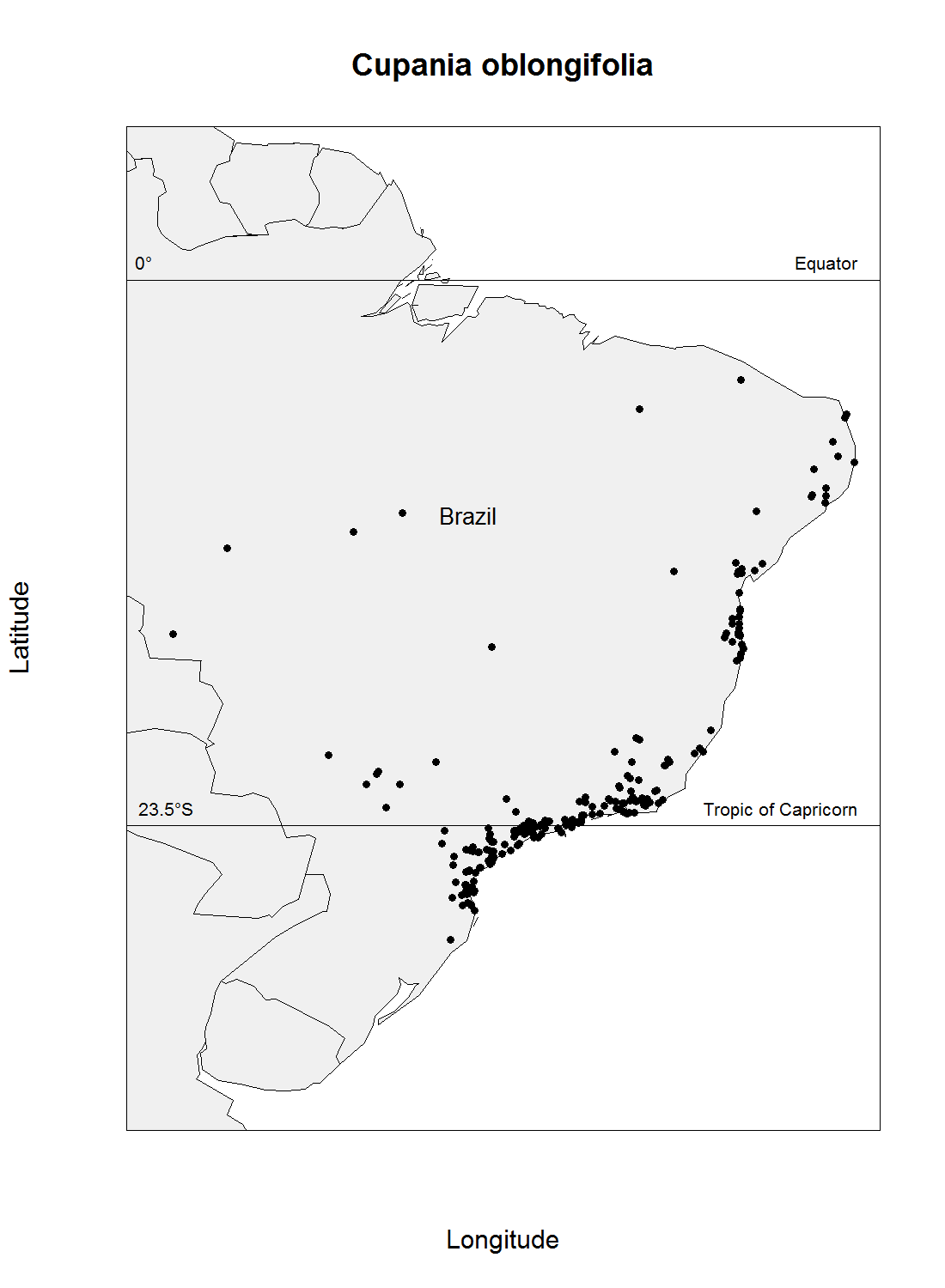 | 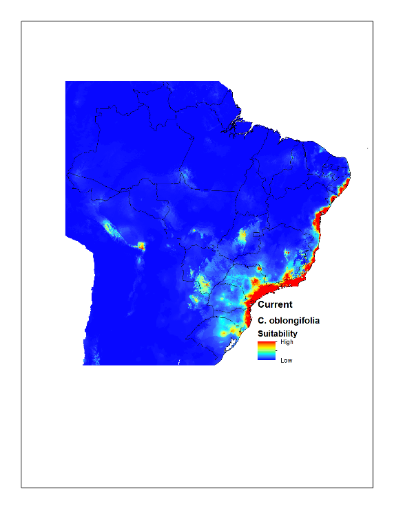 | 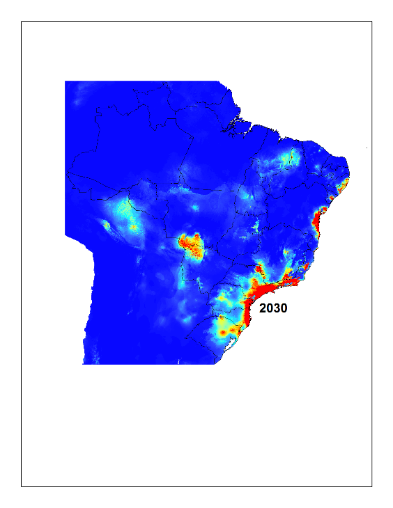 |
| --- | --- | --- |
|  | 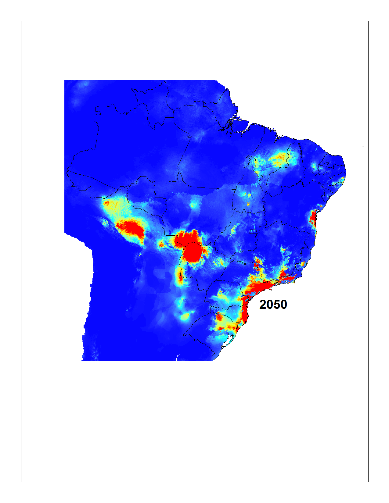 | 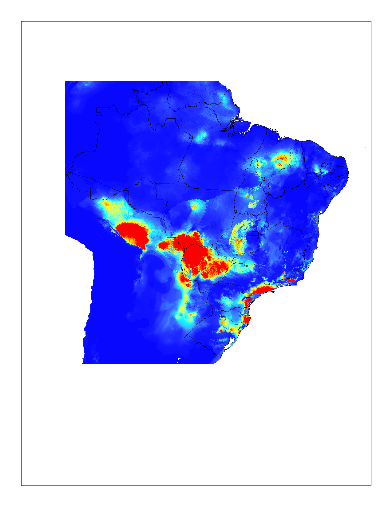 |
| 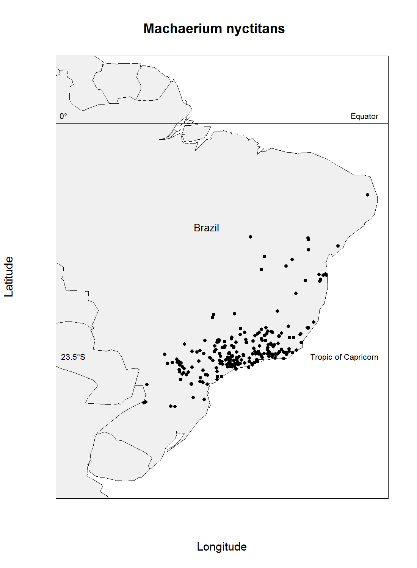 | 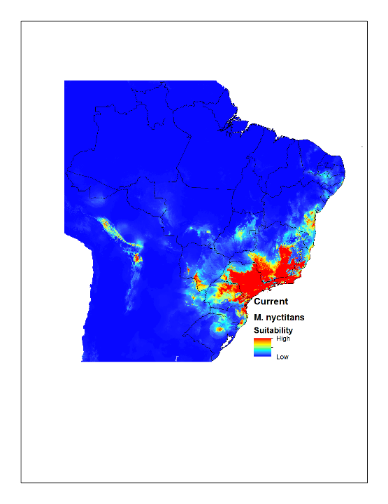 | 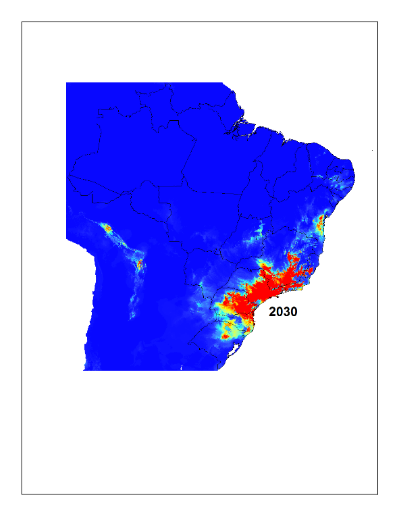 |
|  | 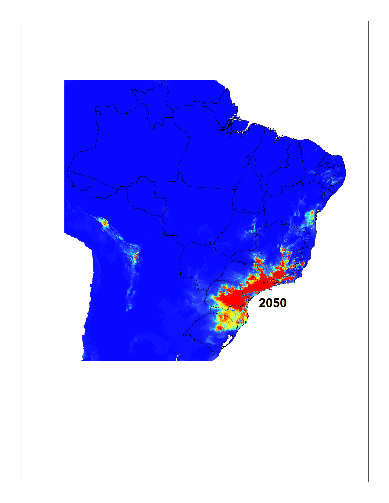 | 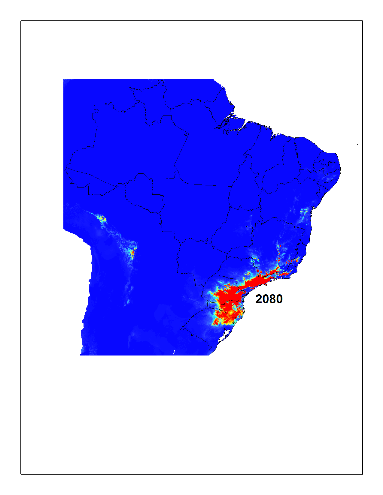 |
| 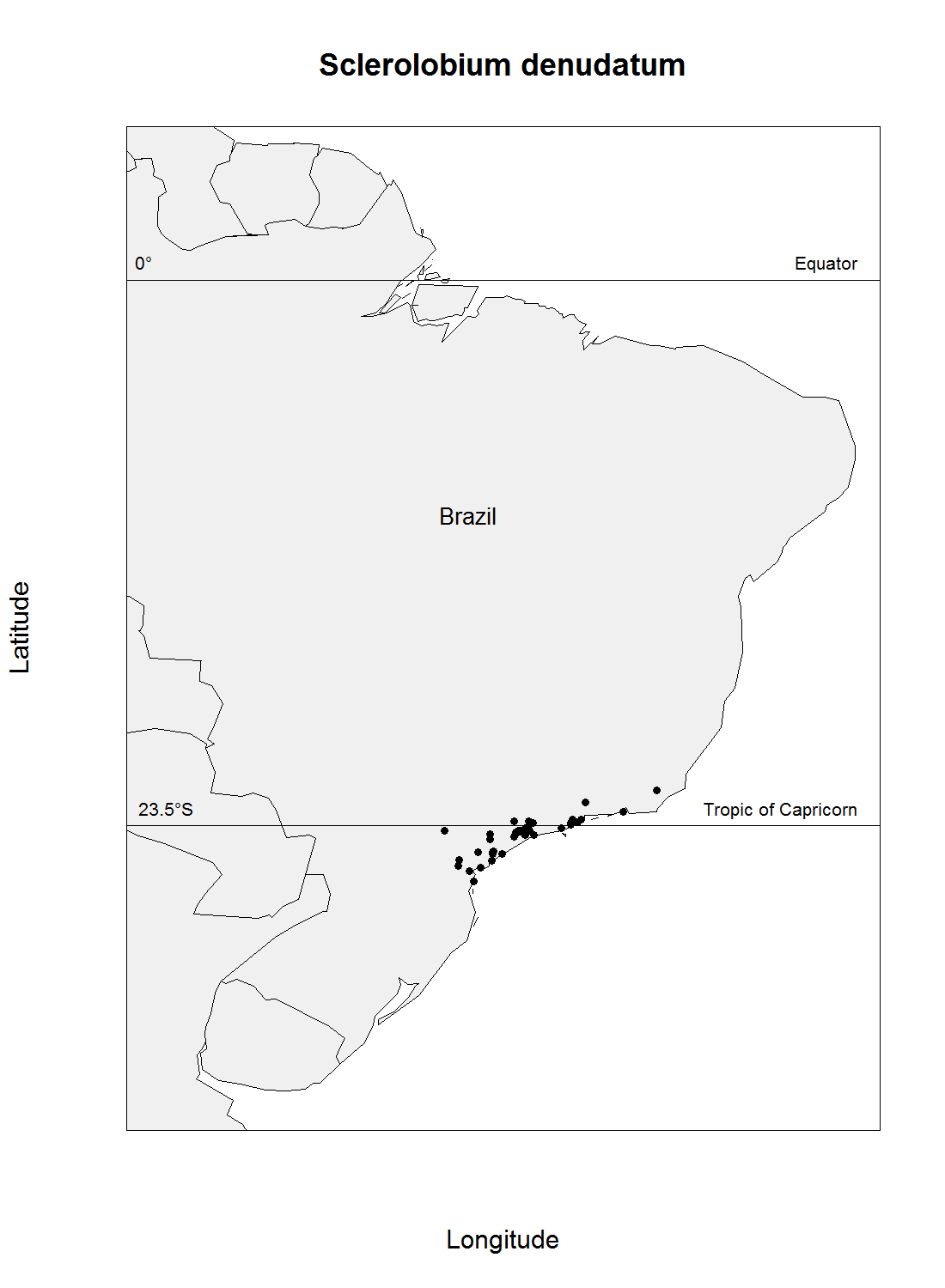 | 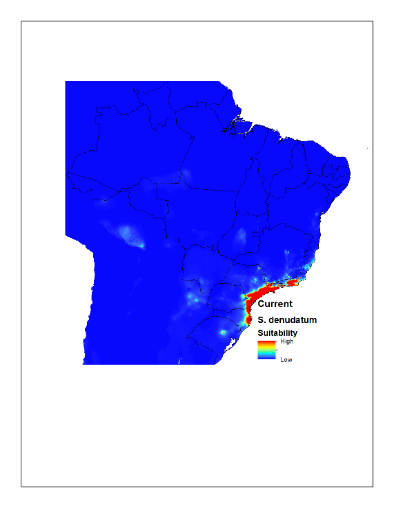 | 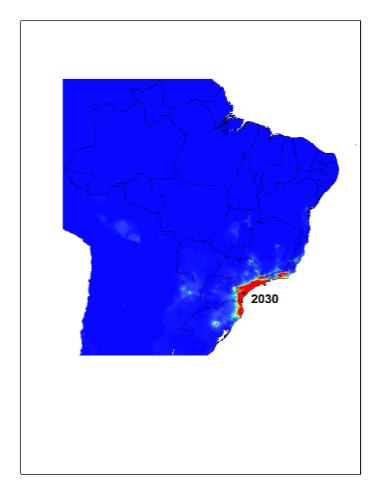 |
|  | 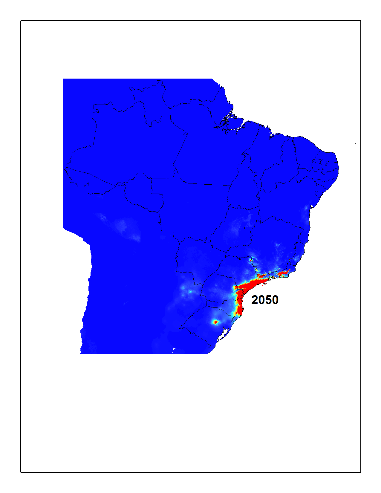 | 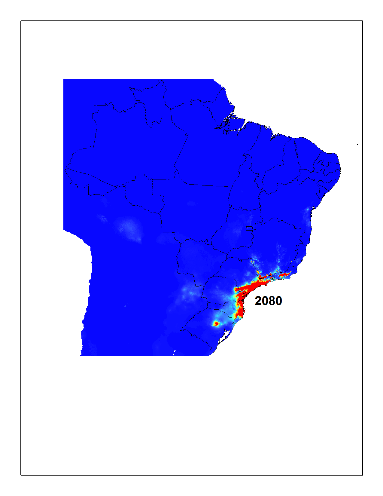 |
| 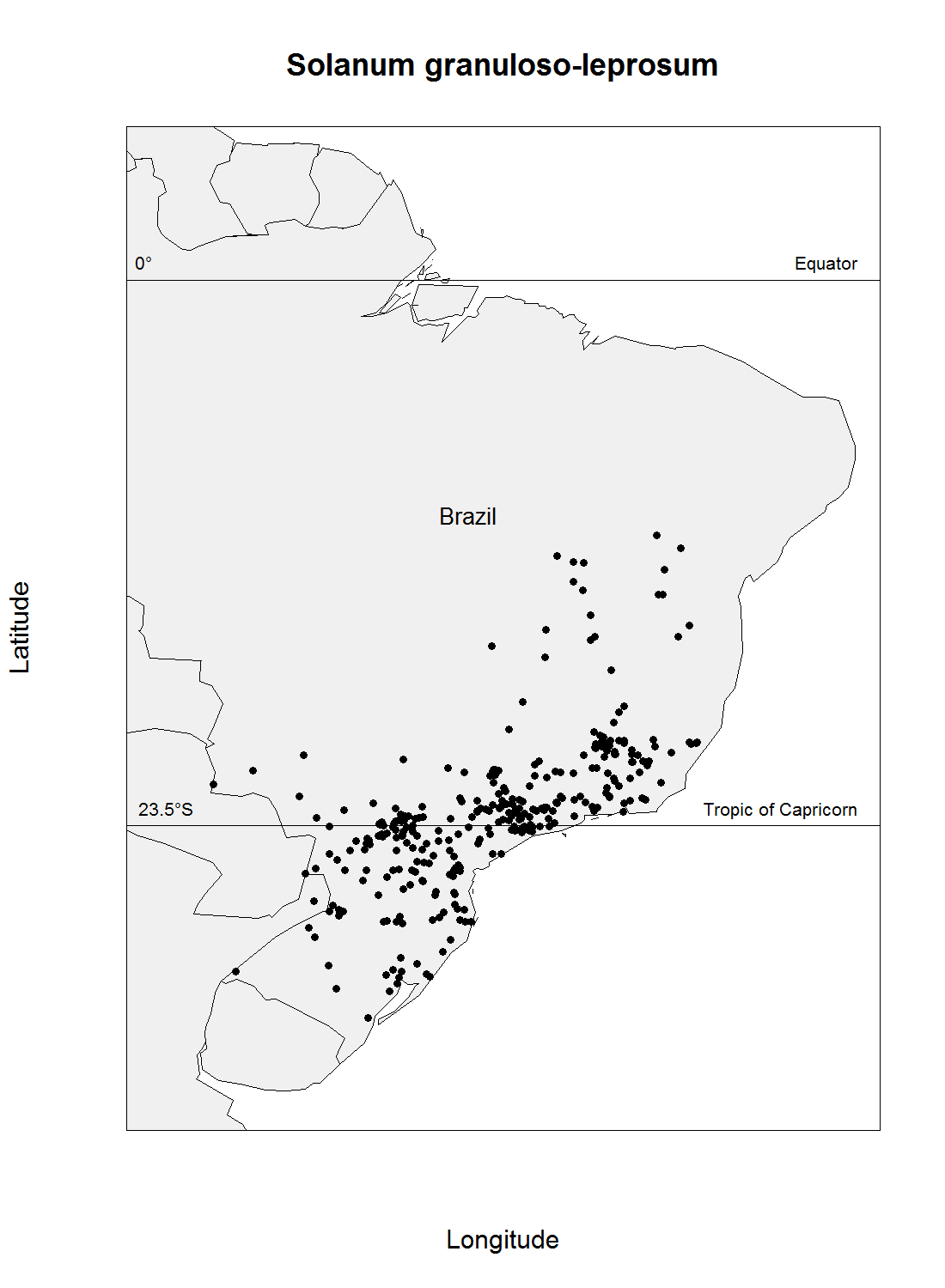 | 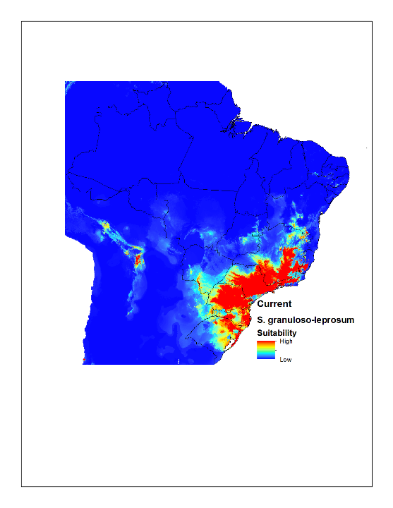 | 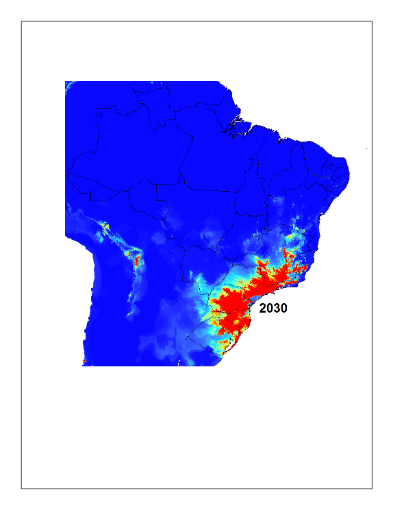 |
|  | 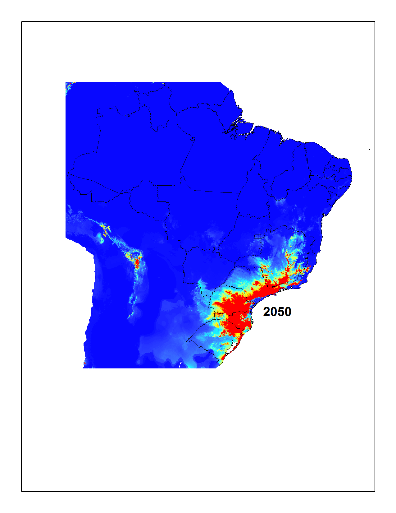 | 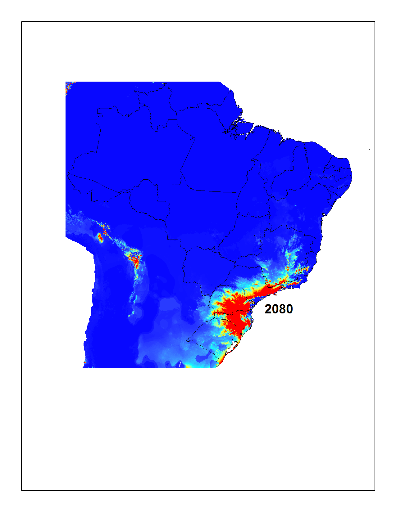 |
| 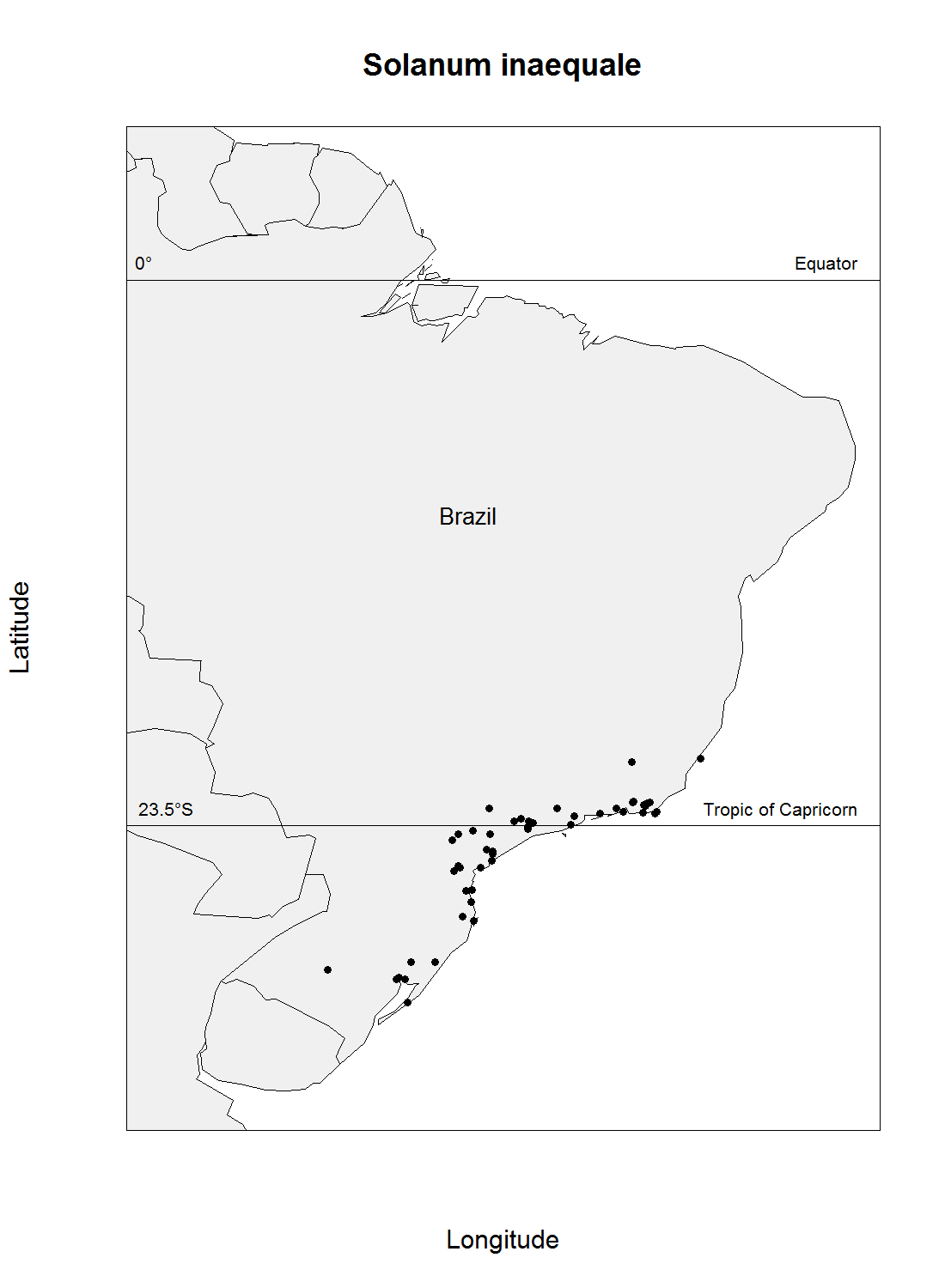 | 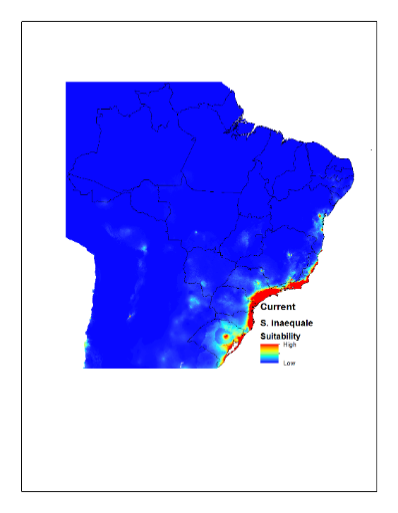 | 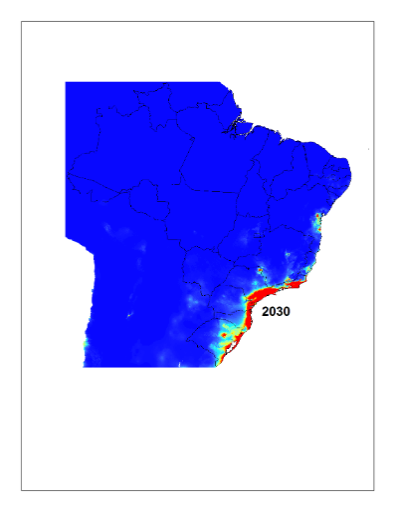 |
|  | 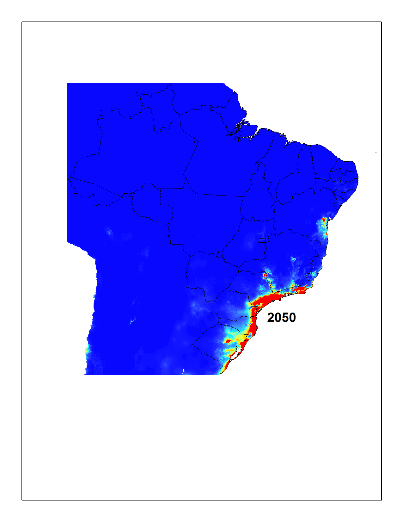 | 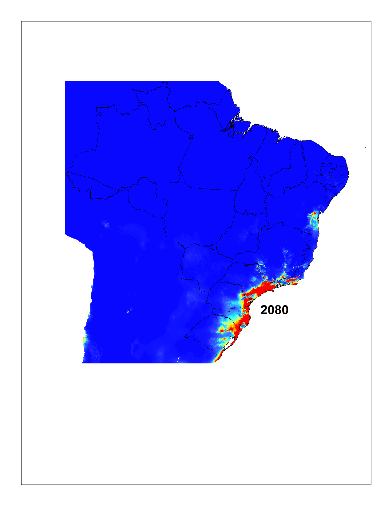 |
| 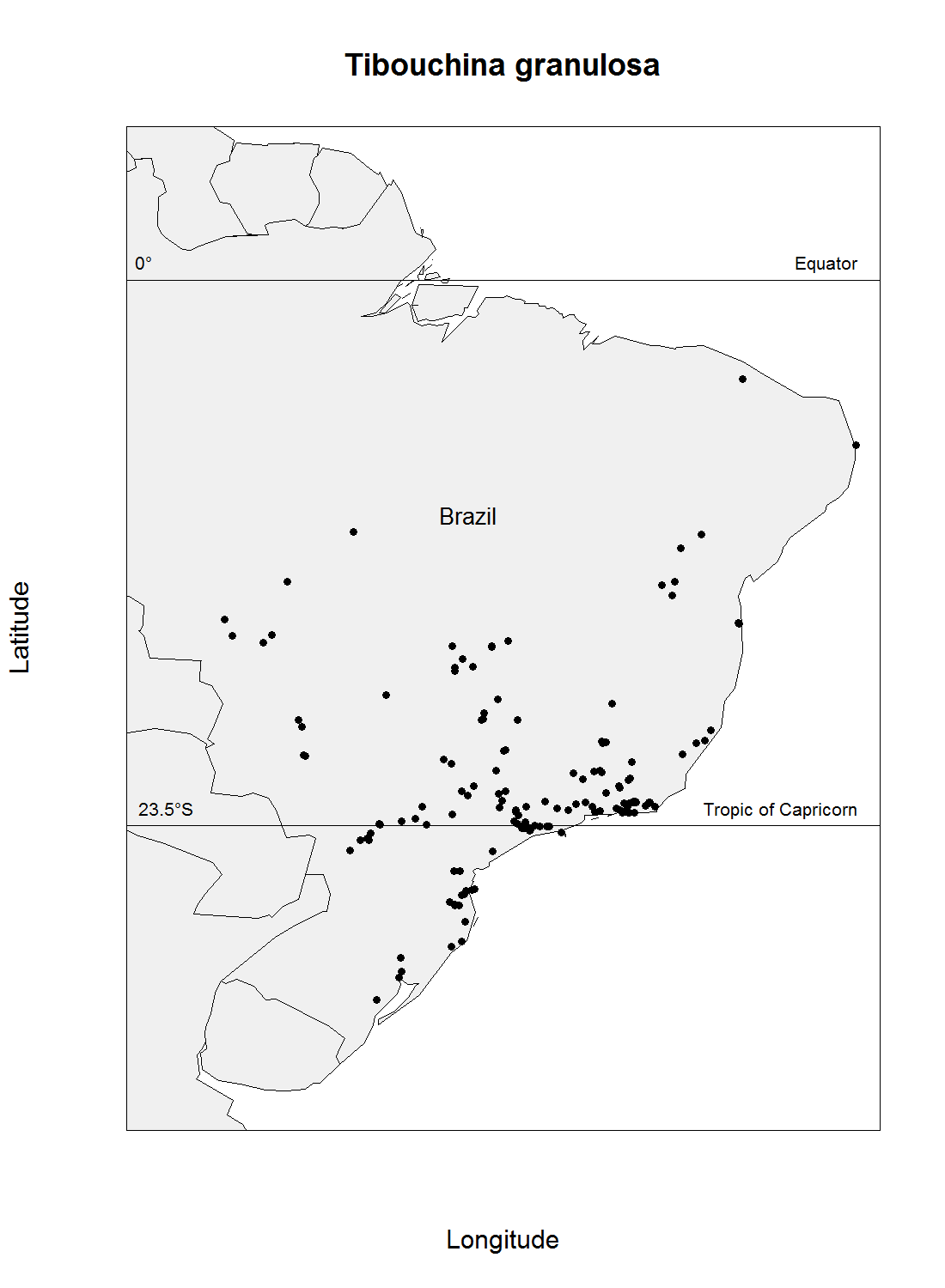 | 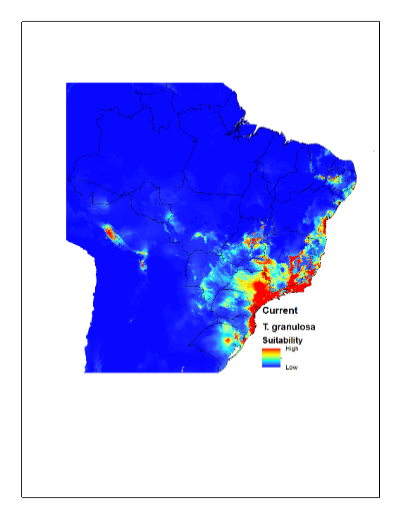 | 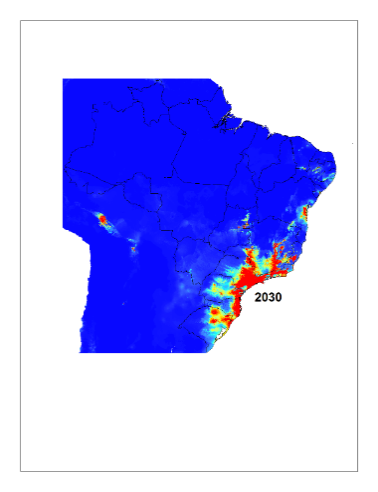 |
|  | 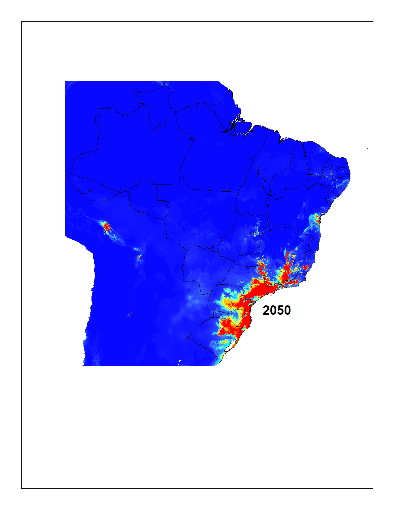 | 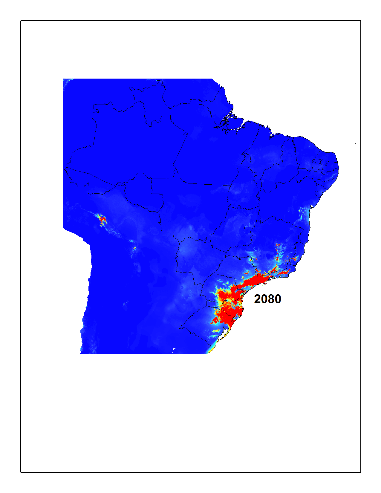 |

S2 Fig. Occurrences reported for *Melipona quadrifasciata* and the seven plant species used as biotic layers and the distributional model obtained for each plant. Models were based only on climatic variables (see Material and Methods for details).
